# Log (*Lactobacillus crispatus*/ *Gardnerella vaginalis*): a new indicator of diagnosing bacterial vaginosis

**DOI:** 10.1080/21655979.2022.2027059

**Published:** 2022-01-18

**Authors:** Tongyang Deng, Anquan Shang, Ying Zheng, Lianzhen Zhang, Hong Sun, Wei Wang

**Affiliations:** a Department of Laboratory Medicine, Tongde Hospital of Zhejiang Province, Hangzhou, P.R. China; b Department of Laboratory Medicine, Shanghai Tongji Hospital, School of Medicine, Tongji University, Shanghai, P.R. China; c Department of Obstetrics and Gynecology, Tongde Hospital of Zhejiang Province, Hangzhou, P.R. China

**Keywords:** Bacterial vaginosis, indicator, lactobacillus crispatus, gardnerella vaginalis

## Abstract

To explore a new marker which can detect bacterial vaginosis (BV) with high sensitivity and specificity quantitatively. According to the Nugent Score, vaginal secretions from study participants were divided into BV, healthy, and BV-intermediate groups. First, we compared the obvious differences and high abundance of bacteria in the three groups using 16S rRNA-sequencing, and screened out candidate markers. Then, quantitative detection of these candidate markers from the three groups was done using real-time reverse transcription-quantitative polymerase chain reaction (RT-qPCR), followed by evaluation of the sensitivity and specificity. Finally, we verified the new markers using clinical cases. *Gardnerella vaginalis, Atopobium vaginae, Lactobacillus, Megasphaera* were screened out by 16S rRNA-sequencing. RT-qPCR data were transformed and analyzed through ROC curves. PCR results for these bacteria were log-transformed using *Lactobacillus crispatus* as the numerator and other BV-related bacteria as the denominator. Four new indicators were found. Of these, log *L. crispatus*/*G. vaginalis* (L/G) <0 was the best indicator. The sensitivity, specificity, positive predictive value, and negative predictive value of our system were 93.5%, 97.2%, 96.6 and 94.6%, respectively. Combination of data for 16S rRNA-sequencing and RT-qPCR revealed four indicators for BV detection. Of these, log L/G < 0 was the best indicator. Creating a molecular-diagnostic system independent of the Nugent Score for BV could have an important impact on the clinical management of BV.

**Abbreviation**: log *L. crispatus*/*G. vaginalis* (logL/G); Bacterial vaginosis (BV); vaginal secretions (VSs); polymerase chain reaction (PCR); rRNA-sequencing (rRNA-seq); real-time reverse transcription-quantitative polymerase chain reaction (RT-qPCR); operational taxonomic unit (OTU); non-metric multidimensional scaling (NMDS); receiver operating characteristic (ROC).

## Introduction

Bacterial vaginosis (BV) is a clinical manifestation caused by a decrease in the abundance of *Lactobacillus* species and microbial imbalance in the vagina. An increase in thin vaginal secretions (VSs) is the main clinical manifestation. The prevalence of BV in women of child-bearing age is 8–40% [1]. Studies have shown that BV is related to premature birth, miscarriage, infertility, premature rupture of membranes, neonatal infection and pelvic inflammation [2,3]. Also, BV may increase the risk of the trichomonad vaginitis, vulvovaginal candidiasis, vulnerability to infection by the human immunodeficiency virus, and various sexually transmitted diseases [4–8], and a high reproductive rate [9,10]. About 10–40% of women with BV may be asymptomatic. Some studies have reported that >50% of pregnant women manifest no symptoms [11,12]. Therefore, the clinical diagnosis of BV may be reliant mainly on laboratory indicators.

The Amsel criteria are used widely to diagnose BV. The presence of three out of four positive criteria indicates that the cause of vaginal complaints is BV: (i) thin, white, yellow, homogeneous discharge; (ii) clue cells on microscopy; (iii) pH of vaginal fluid >4.5; (iv) release of a ‘fishy’ odor upon addition of 10% potassium hydroxide solution. However, application of the Amsel criteria can be subjective (e.g., judging a fishy smell) and is, therefore, limited.

Another way to diagnose BV is to use the Nugent Score: a Gram stain scoring system for vaginal swabs first proposed in 1991 [13]. A score from 0 to 10 is obtained from combining three other scores. A score of 0–3 is considered ‘negative’ for BV, 4–6 is considered ‘indeterminate’ for BV, and a score of 7+ is considered to indicate BV. However, use of the Nugent Score requires highly trained and experienced staff. Also, reliance on morphology for identifying bacteria is subjective, and can lead to mis-identification of bacteria. For example, *Lactobacillus* species with indeterminate staining can be mistaken for *Gardnerella vaginalis* or other Gram-variable bacilli [14,15]. During the past decade, molecular diagnostic tools have increased in importance to aid diagnoses [16–18], and have led to various methods to diagnose BV [19].

Major studies using polymerase chain reaction (PCR) methods have been employed to directly detect bacteria in VSs in relation to BV. About 35 bacterial species have been implicated in BV, including *G. vaginalis, Atopobium vaginae, Porphyromonas asaccharolytica*, various genera (*Megasphaera, Sneathia, Prevotella, Peptostreptococcus*) and bacterial vaginosis-associated bacterium (BVAB)1–3 [20–25]. Some commercial reagent kits can be used to diagnose BV through combined detection of *A. vaginae, G. vaginalis, Megasphaera* species (types 1 and 2), BVAB (type 1 and/or type 2) and *Lactobacillus* species. Other some reagent kits use single quantitative detection of *G. vaginalis* to diagnose BV. However, which may make the detecting indicator more complicated or simpler, especially, single bacterial species detection may ignore imbalanced vaginal flora. Moreover, the quantitative detection of bacteria will also be affected by secretion appearance, volume, and other uncontrollable factors [26–28].

Compared with PCR methods, although 16S rRNA-sequencing (16S rRNA-seq) technology cannot detect vaginal flora quantitatively, it reflects the relative abundance and different varieties of vaginal microflora in BV patients. In this study, we propose to use 16S rRNA sequencing technology to screen out the differential bacteria in vaginal secretions of healthy people and BV patients, and to diagnose BV by comparing the ratio of beneficial bacteria (e.g. Lactobacillus) to harmful bacteria (e.g. Gardnerella vaginalis), which can better reflect the thinking of flora balance, while avoiding the problem of inaccurate absolute quantification of bacteria in vaginal secretions; and to find out the sensitivity and specificity from the differential bacteria The most optimal index to reduce the number of molecular targets needed to diagnose BV in practice, and finally to achieve the purpose of saving test cost and accurately diagnosing BV.

## Materials and methods

### Ethical approval of the study protocol

The study protocol was approved (2021056) by the Ethics Committee of Tongde Hospital of Zhejiang Province, China.

**Grouping the screening step and establishing inclusion and exclusion criteria**: the inclusion criteria were women: (i) with a regular menstrual cycle: the menstrual cycle lasts from 21 to 35 days, lasts from 2 to 8 days, and the menstrual volume is 20 to 60 ml; (ii) aged 18–46 years; (iii) who were not pregnant; (iv) not using vaginal contraceptives; (v) without *Candida* species, *Neisseria gonorrhoeae, Trichomonas* species or other pathogens in their VSs; (vi) who had not used antibiotics in the previous 3 months; (vii) No sexual intercourse within 24 hours. (viii) To rule out ovarian and uterine related tumors.

Women were divided into the healthy-volunteer (N) group, BV intermediate (M) group and BV (B) group.

For group N, VSs were collected from healthy women. These women had undergone physical examination in our hospital previously. Their Nugent Score was 0–3, the pH of the VS was 3.8–4.5, and *Candida* species and *Trichomonas* species were absent. The cleanliness degree was I–II. The VS had a normal appearance, without a fishy odor, and vaginal itching/burning sensations were absent.

For group B, vaginal secretions were collected from outpatients in the gynecology department of our hospital. *Candida* species and *Trichomonas* species were not detected. The Nugent Score was 7–10. The VS met two items of the Amsel criteria (clue cells were present and pH >4.5).

For group M, VSs were collected from outpatients in the gynecology department of our hospital. *Candida* species and *Trichomonas* species were absent, and the Nugent Score was 4–6.

The enrollment criteria for clinical verification differed according to the group. For group N, the criteria were identical to the upper inclusion standard. For group B, the Nugent Score was 7–10, clue cells were present, and pH >4.5. *Candida* and *Trichomonas* species and other pathogens were not eliminated.

### Clinical validation phase case enrollment criteria

Group N: pregnancy was not excluded, other criteria were the same as in the screening phase. Group B: Nugent score 7–10 and positive clue cells or vaginal discharge PH > 4.5, age 18–46 years, no history of sexual intercourse for 24 hours. To verify the accuracy of the index in a complex setting, no other exclusion criteria were set.

### Clinical specimens

A gynecologist used a sterile swab to wipe-off secretions outside the vulva. Then, two sterile nylon vaginal swabs were placed into the lower one-third of the vagina. and sent for analyses within 1 h. In one sample, 0.5 mL of physiologic (0.9%) saline was added to measure the degree of cleanliness, presence of *Candida* species or *Trichomonas* species, other routine tests, and Gram staining for the Nugent Score. Another sample was immediately stored at − 80°C for DNA extraction, 16S rRNA sequence and RT qPCR.

### DNA extraction

DNA was extracted using a commercial reagent kit from Hunan Shengxiang Biological Technical (Hunan, China) according to manufacturer instructions, The vaginal secretion specimens containing the nucleic acids to be extracted were lysed by the lysis solution to lyse the cells, and the DNA was obtained by washing, elution and purification processes using magnetic beads that specifically recognize and efficiently bind to the DNA molecules and adsorb the beads to the tube walls using a magnetic separator. Then nuclear purity was measured using Qubit™ 2.0 (Thermo Fisher, Waltham, MA, USA). Extracted DNA was analyzed by a spectrophotometer at wavelengths of 260 nm (excitation) and 280 nm (emission). DNA was stored at −40°C.

### 16S rRNA-seq

The V4 variable area of bacteria (primer: 515 F-806 R) was amplified and sequenced through a HiSeq™ platform (Illumina, San Diego, CA, USA). First, the purity and concentration of the DNA in the sample was measured by agarose gel electrophoresis. Samples were placed in a centrifuge tube and diluted to 1 ng/μL in sterile water. Using diluted DNA as a template and specific primers with barcodes, amplification was carried out by Phusion® High-Fidelity PCR Master Mix with GC Buffer (New England Biolabs, Ipswich, MA, USA) and high-efficiency enzymes.

PCR outcomes were detected with 2% agarose gel by electrophoresis. Strips of size 400–450 bp were recycled by gel kits (Qiagen, Hilden, Germany). A TruSeq® DNA PCR-Free Sample Preparation kit (Illumina) was used to construct DNA libraries. The constructed libraries were detected quantitatively by Qubit and RT-qPCR, and the relevant libraries were sequenced on the HiSeq platform.

### Data manipulation

Data were obtained through splicing and filtering of primary data gained by sequencing. These data were used to analyze operational taxonomic unit (OTU) clusters and species classification. We obtained information on the species, abundance, and distribution of bacteria. Multiple sequence comparisons were performed on OTUs and phylogenetic trees were constructed, and further differences in community structure were obtained for different samples and subgroups, which were presented by downscaling plots such as PCoA, PCA and NMDS.

### Fluorescence RT-qPCR

DNA extracted from VSs was used to detect 14 common pathogens. We used a 7500 Real-time PCR system (Applied Biosystems, Foster City, CA, USA) and the TaqMan™ Gene Expression Assays kit (Thermo Fisher). For the latter, the total reaction volume was 20 µL (20 × TaqMan Gene Expression Assay (1.0 μL), 2 × TaqMan Gene Expression Master Mix (10.0 μL), DNA template (4.0 μL), double-distilled H_2_O (5.0 μL). The PCR parameters were: 50°C for 2 min, 95°C for 10 min in pre-denaturation, 95°C for 15 s in denaturation, 60°C for 1 min in annealing extension, in a total of 40 cycles.

## Results

### Basic information of patients

Many doctors utilize the Amsel criteria for diagnosing BV. However, application of the Amsel criteria can be subjective and is, therefore, limited. The Nugent Score, a Gram stain grading system for vaginal swabs initially introduced in 1991, is another method for BV diagnosis. A well-versed team is required to employ the Nugent Score, though. Furthermore, relying just on morphology to identify bacteria is prone to error due to the subjectivity involved. This research aims to discover a new bacterial vaginosis quantitative marker with excellent sensitivity and specificity. From April to September 2020, a total of 109 women were enrolled. Group N comprised 39 cases, and their mean age was 39.6 ± 7.15 years. Group B was composed of 37 cases of mean age 38.9 ± 8.03 years. Group M comprised 33 cases of mean age 37.9 ± 6.03 years. There was no significant difference according to age (ANOVA) among the three groups (P = 0.623). Clue cells were detected in group B (23/37, 62.16%) but not in the other two groups.

### 16S rRNA-seq

**Figure 1.**
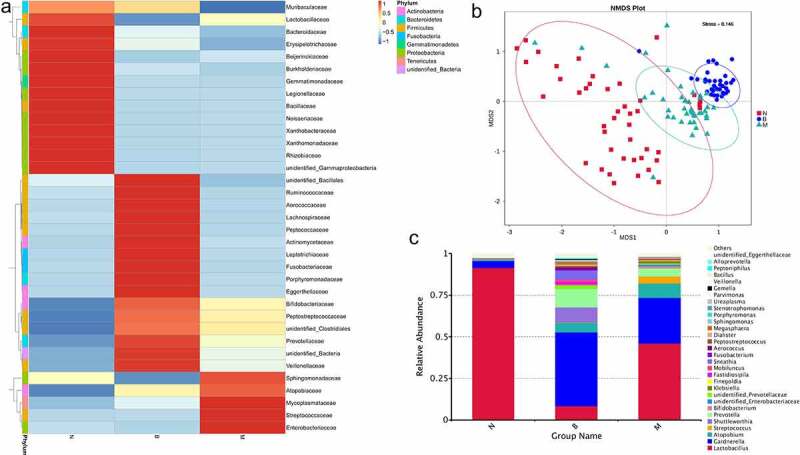
The detection of differential abundance of the healthy, BV and BV intermediate groups by 16S rRNA sequencing.

Heatmaps were drawn for different groups to show the genus level (Figure 1(a)). There were obvious differences in the genus level among the three groups. *Lactobacillus* species were in high abundance in group N. Bacteria of genera *Gardnerella, Mobiluncus, Megasphaera, Sneathia, Peptostreptococcus, Veillonella, Prevotella, Gemella* and *Mycoplasma* showed high abundance in group B. Group M showed high abundance of bacteria of genera *Atopobium, Klebsiella, Enterobacteriaceae, Streptococcus,Ureaplamsa* and *Sphingomonas*. Bacteria of genera *Lactobacillus, Gardnerella, Atopobium, Prevotella, Shuttleworthia* and *Streptococcus* were the most abundant among the three groups (Figure 1(c)). The abundance of *Lactobacillus* species in group N was >75%. The abundance of Gardnerella species in group B was ~50%. NMDS for analyses of species diversity revealed remarkable differences among the three groups (Figure 1(b)). Group N and group B could be distinguished readily. whereas group M lay intermediate between group N and B. The stress was 0.146. Each point in (Figure 1(b)) represents a sample, and the distance between points represents the degree of difference. Stress <0.2 indicates that NMDS may reflect the degree of difference in different samples accurately.

A: Heatmap of bacteria at the genus level.B: The analysis of NMDS based on OUT levelC: Relative abundance of the top-30 bacteria at the genus level.

### PCR results

We combined 16S rRNA-seq results with data for microorganism species that could be detected using commercial kits. *Lactobacillus crispatus, Lactobacillus iners, Lactobacillus jensenii, Lactobacillus gasseri, G. vaginalis, Atopobium vaginae*, BVAB-2, *Megasphaera*-1, *Megasphaera*-2, *Prevotella bivia, Mobiluncus curtisii, Mobiluncus mulieris, Mycoplasma hominis*, and *Ureaplasma urealyticum* were the main species detected in 109 VSs by PCR. The test results were logarithm-converted (if a test result was negative, a value of 1 was used), followed by analyses by the Spearman rank test. Results indicated that only *L. crispatus* was negatively associated with BV grade among the four species of *Lactobacillus*, with r = −0.651. Other microflora had a significant (P < 0.05) positive relationship with BV grade, with a range of 0.206 to 0.748. *G. vaginalis* had the highest positive correlation (r = 0.748) (Table 1).

**Table 1.** The analysis of the correlation between 14 common vaginal microorganisms and BV

	***Lactobacillus*** ***crispatus***	***Lactobacillus*** ***iners***	***Lactobacillus*** ***jensenii***	***Lactobacillus*** ***gasseri***	***Gardnerella*** ***vaginalis***	***Atopobium*** ***vaginae***	**BVAB-2**
**Spearman coefficient**	−0.651	−0.107	0.032	−0.107	0.748	0.57	0.63
***P***	<0.01	0.266	0.741	0.266	<0.01	<0.01	<0.01
	***Megasphaera-1***	***Megasphaera-2***	***Prevotella*** ***bivia***	***Mobiluncus*** ***curtisii***	***Mobiluncus*** ***mulieris***	***Mycoplasma*** ***hominis***	***Ureaplasma*** ***urealyticum***
**Spearman coefficient**	0.578	0.536	0.209	0.422	0.266	0.513	−0.098
***P***	<0.01	<0.01	0.029	<0.01	0.005	<0.01	0.313

**Figure 2.**
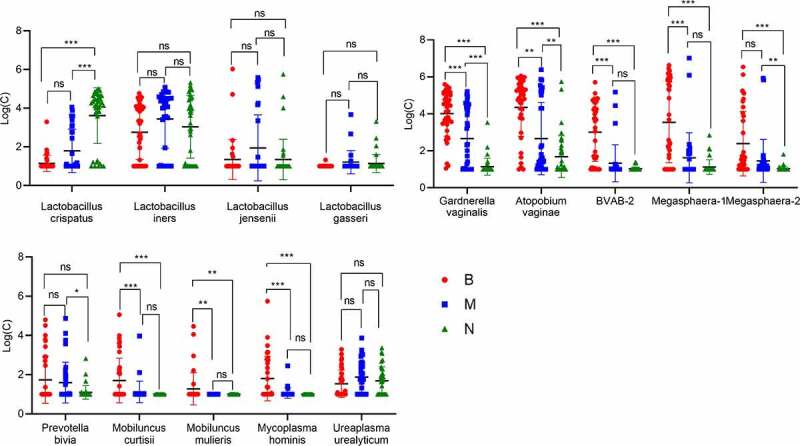
Comparison of RT-qPCR results for bacteria in groups B, M and N. ns:p > 0.05,*:P < 0.05, **:P < 0.01, ***:P < 0.001.

Test results were log-transformed. Based on the convenience of drawing and because zero cannot be log-transformed, if the test result was lower than the limit of detection, we used a value of 10. We undertook the Kruskal–Wallis test using α = 0.05 as the detection level (Figure 2). *L. crispatus, G. vaginalis, A. vaginae*, BVAB-2, *Megasphaera*-1, *Megasphaera*-2, *M. curtisii, M. mulieris*, and *M. hominis* in group B and group N were present in significant amounts. Considering that there are many negative results of Mobiluncus and Mycoplasma hominis, and some positive results were mostly less than 100 copies, and the sensitivity, repeatability as well as accuracy were not ideal in our experiment. Therefore, the top-five most abundant bacterial species were selected for further analyses.

**Figure 3.**
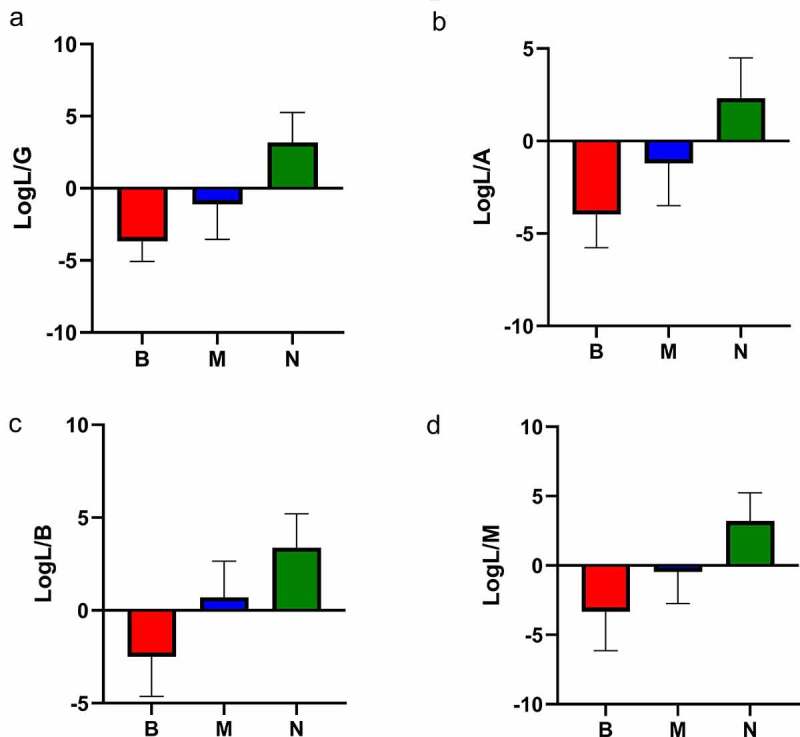
Indicators of log L/G, log L/A, log L/B and log L/M among the three groups.

**Figure 4.**
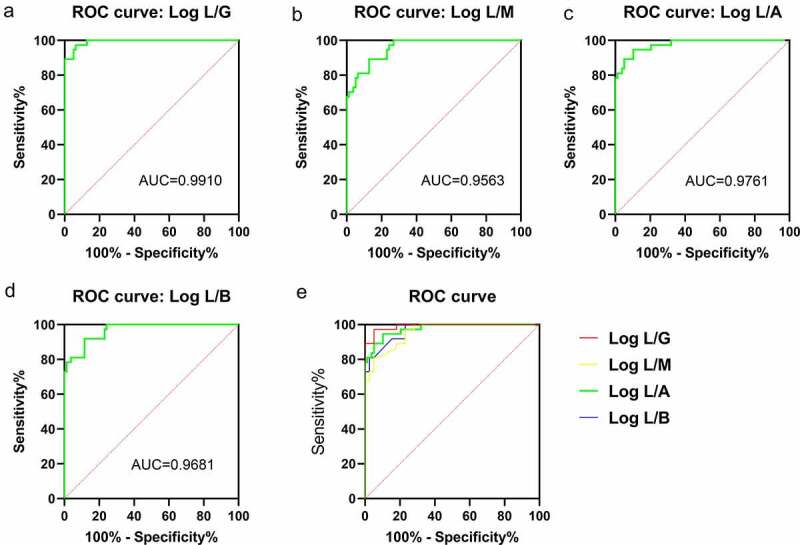
ROC curves for log L/G, log L/A, log L/B and log L/M used for a BV diagnosis.

- *crispatus* was negatively related (r = −0.651) with the BV grade, whereas *G. vaginalis, A. vaginae*, BVAB-2, *Megasphaera*-1, and *Megasphaera*-2 had a positive association with BV. The PCR results for these bacteria were log-transformed using *L. crispatus* as the numerator and other BV-related bacteria as the denominator. Four new indicators were found: log L/G = log (*L. crispatus*/*G. vaginalis*), log L/A = log (*L. crispatus*/*A. vaginae*), log L/B = log (*L. crispatus*/BVAB2), and log L/M = log (L. *crispatus/Megasphaera* 1/2). The denominator cannot be zero, and zero cannot be log-transformed so, if the test result was lower than the limit of detection, we used a value of 1 (Figure 3). Figure 3 shows that group B and group N were well differentiated through use of the four indicators stated above. Using the Nugent Score as the standard classification and the four indicators as the threshold value, ≥0 was diagnosed as ‘BV-negative’, whereas <0 was diagnosed as ‘BV-positive’. The sensitivity, specificity, positive predictive value, and negative predictive value for each indicator for diagnosing BV are shown in (Table 3). After analyses using a receiver operating characteristic (ROC) curve and comparing it with each indicator, log L/G was selected as the best indicator for diagnosing BV (Figure 4).

**Table 2.** The case characteristics analysis of the BV-negative and BV-positive groups

Item	BV-negative	BV-positive
Patients (number)	103	92
*Candida*-positive (number)	2	12
Age (years)	33.1 ± 8.1	34.4 ± 9.9
Cleanliness of secretions	I–II	III–IV
pH of secretion	4.0 ± 0.2	4.9 ± 0.3
Nugent Score	0–3	7–10
Cystitis (number)	31	50
Cervicitis (number)	2	1
Pelvic inflammation (number)	4	3
Uterine non-inflammatory disease (number)	21	11
Pregnancy (number)	32	8
Physical examination (number)	8	9
Other (number)	5	10

**Table 3.** Use of the four indicators to evaluate a BV diagnosis

New indicator		BV positive* case	BV negative* case	Sensitivity	False- positive rate	Positive predicted value	Specificity	False-negative rate	Negative predicted value	Consistency	Youden Index	Kappa
L/G	BV positive	36	3	97.3%	7.5%	92.3%	92.5%	2.7%	97.4%	94.81%	89.80%	0.896
	BV negative	1	37									
L/A	BV positive	35	7	94.6%	17.5%	83.3%	82.5%	5.4%	94.3%	88.31%	77.09%	0.767
	BV negative	2	33									
L/M	BV positive	30	3	81.1%	7.5%	90.9%	92.5%	18.9%	84.1%	87.01%	73.58%	0.739
	BV negative	7	37									
L/B	BV positive	30	2	81.1%	5.0%	93.8%	95.0%	18.9%	84.4%	88.31%	76.08%	0.765
	BV negative	7	38									
L/G&	BV positive	86	3	93.5%	2.8%	96.6%	97.2%	6.5%	94.6%	95.50%	0.907	0.910
	BV negative	6	106									

*BV diagnosis was made using the Nugent score.

#BV diagnosis was made using logL/G, <0 diagnosed as BV positive, ≥0 indicates BV negative.

&Compare two BV diagnostic indexes logL/G with Nugent score.

### Clinical verification of the indicator log L/G

From December 2020 to February 2021, case information during indicator verification stage was presented in (Table 2).

We used log L/G on VSs collected from 103 cases classified as not suffering from BV and 92 cases suffering from BV. When using log L/G < 0 as the criterion, test results demonstrated a sensitivity of 93.5%, specificity of 97.2%, positive predictive value of 96.6, and negative predictive value of 94.6% (Table 3).

## Discussion

The stability of the vaginal microbiota is influenced by the menstrual cycle, sex life, and other factors [29,30]. Therefore, different studies may elicit different results when studying the vaginal microbiota. Our 16S rRNA-seq results indicated that the most abundant bacteria were from the genus Lactobacillus in group B, and G. vaginalis in group N, data which are similar to those in the literature [31]. Also, our 16S rRNA-seq results were consistent with our PCR results. The abundance of sequencing results was not further analyzed at the species level in this study because 16S rRNA sequencing technology may have large errors at the species level. Subsequently, RT-qPCR was employed to detect Lactobacillus species.

Several studies have stated that *G. vaginalis* participates in the morbidity and immune-modification in women suffering from BV. Also, irrespective of whether culture or molecular diagnostics are employed, the sensitivity of detection of *G. vaginalis* is ≤100%, whereas the specificity is only 50% [3], Because G.vaginalis was present in the vaginal secretions of both BV and non-BV females [32,33], The difference may be in the significantly higher number of Gardenella in BV patients compared with healthy women. For women with BV symptoms, using 5 × 10^5^ as the threshold value, BV could be diagnosed thanks to *G. vaginalis* detection using the Affirm VP Microbial Identification System (Beckton Dickinson, Franklin Lakes, NJ, USA). The sensitivity of that system is 90% and specificity is 97% [34]. Cartwright and colleagues proposed combining detection of *A. vaginae*, BVAB-2, and *Lactobacillus* species with that of *Megasphaera*-1. Considering the specificity, *G. vaginalis* was excluded, and they obtained a sensitivity of 96.7% and specificity of 92.2% for patients with clinical symptoms [35]. Hilbert and coworkers suggested combining detection of *G. vaginalis* with that of *Megasphaera*-1 and *Megasphaera-*2 besides *L. crispatus* for diagnosing BV, and obtained a sensitivity of 92% and specificity of 95% [36].

After RT-qPCR, we found that other common *Lactobacillus* species have no relationship with BV besides *L. crispatus*. Data for *A. vaginae*, BVAB-2, *G. vaginalis*, and *Megasphaera-*1 and 2 were combined with the Nugent Score and vaginal-flora characteristics of BV. Then, the data were log-transformed into four new indicators: log L/G, log L/B, log L/M, and log L/A. After comparison of these four indicators, log L/G < 0 was found to be the best indicator for a BV diagnosis with a sensitivity of 97.3% and specificity of 92.5%. During clinical verification of this indicator, the sensitivity and specificity was 93.5% and 97.2%, respectively, which are similar to those for some commercial reagent kits for diagnosing BV through detection of various bacteria types [37]. However, only two bacteria species were examined in this study.

At a similar sensitivity and specificity, compared with single detection of *G. vaginalis* or combining data for numerous bacteria, log L/G is more appropriate for the flora imbalance seen in BV. Compared with other bacteria associated with BV, *L. crispatus* and *G. vaginalis* have a higher relative abundance and copies in VSs, which makes it better linear range and repeatability in the process of detection. The ratio of abundance of *L. crispatus* to that of *G. vaginalis* was used, so the amount of VS sampled had little effect on log L/G. Compared with the Nugent Score as the ‘gold standard’, log L/G can be quantified accurately, monitored continuously, and avoids the subjectivity inherent in the morphologic assessment of bacteria.

In some VSs, bacterial recognition based on morphology can be difficult, and the Nugent Score recognizes bacteria only from the genera *Lactobacillus, Gardnerella*, and Mobiluncus, while other bacteria causing dysbacteriosis are not distinguished [38] Therefore, using this evaluation system, these patients are easily classified into BV intermediate type, which hampers the clinical diagnosis and treatment. Using log L/G for detection of the M group elicits the same problem in this study. In the intermediate group, there were both cases with LogL/G > 0 and cases with LogL/G < 0. Some of the cases in the intermediate group were in the transitional stage of dysbiosis, but a small number of cases could be further differentiated into the healthy or BV group, which needs to be combined with more clinical evidence such as clinical presentation and patient outcome. Thus, this study still needs to be expanded and further studies are needed to verify the clinical value of LogL/G in identifying intermediate groups.

## Conclusions

Combination of data for 16S rRNA-seq and RT-qPCR revealed four indicators for BV detection. Of these, log L/G < 0 was the best indicator. Creating a molecular-diagnostic system independent of the Nugent Score for BV could have an important impact on the clinical management of BV.

## Highlights

- logL/G can better measure present vaginal microecology.
- logL/G is quantitative, continuous, and reproducible.
- logL/G has a strong linear range, sensitivity and specificity.
- log L/G may be utilized to detect BV early.
- log L/G can be used as a novel marker for early diagnosis of BV.
